# *In Vivo* Evaluation of the Anti-Schistosomal Potential of Ginger-Loaded Chitosan Nanoparticles on *Schistosoma mansoni*: Histopathological, Ultrastructural, and Immunological Changes

**DOI:** 10.3390/life12111834

**Published:** 2022-11-09

**Authors:** Mona M. El-Derbawy, Hala S. Salem, Mona Raboo, Ibrahim R. Baiuomy, Sana A. Fadil, Haifa A. Fadil, Sabrin R. M. Ibrahim, Walaa A. El Kholy

**Affiliations:** 1 Department of Medical Parasitology, Faculty of Medicine, Al-Azhar University, Cairo 11751, Egypt; 2 Department of Immunology and Parasitology, Theodor Bilharz Research Institute, Giza 12411, Egypt; 3 Department of Natural Products and Alternative Medicine, Faculty of Pharmacy, King Abdulaziz University, Jeddah 21589, Saudi Arabia; 4 Department of Clinical and Hospital Pharmacy, Faculty of Pharmacy, Taibah University, Almadinah Almunawarah 30078, Saudi Arabia; 5 Preparatory Year Program, Department of Chemistry, Batterjee Medical College, Jeddah 21442, Saudi Arabia; 6 Department of Pharmacognosy, Faculty of Pharmacy, Assiut University, Assiut 71526, Egypt

**Keywords:** *Schistosoma mansoni*, ginger, Zingiberaceae, praziquantel, anti-schistosomal, chitosan nanoparticles

## Abstract

Chemotherapy is the most widely advocated method of Schistosome control. However, repeated chemotherapy leads to the emergence of drug-resistant *Schistosoma* strains. Therefore, efforts to find alternative drugs, especially those of natural origin, have risen globally. Nanoparticles (NPs) have received special interest as efficient drug delivery systems. This work aimed to investigate the anti-schistosomal potential of *Zingiber officinale* (ginger, Zingiberaceae)-loaded chitosan nanoparticles (GCsNPs) on *Schistosoma mansoni* experimentally infected mice that were exposed to 80 ± 10 cercariae/mouse. The study groups are: (G1) negative control; (G2) positive control; (G3) praziquantel in a dose of 500 mg/kg/day for two consecutive days; (G4) ginger in a dose of 500 mg/kg treated; (G5) chitosan nanoparticles in a dose 3 mg/kg (G6) GCsNPs in a dose 250 mg/kg; and (G7) GCsNPs in a dose 500 mg/kg. The anti-schistosome potential was assessed using histopathological scanning electron microscopically and immunological parameters. The results showed that there was a significant decrease in cellular granuloma count (*p* < 0.05) and granuloma diameter (*p* < 0.001) in all infected treated mice groups, in comparison to the infected non-treated group with the highest reduction in both G3 and G7. SEM of *S. mansoni* adult worm recovered from G3 showed mild edema of oral and ventral suckers with some peeling and blebs around them, while that recovered from G7 showed abnormal oedematous oral and retracted ventral sucker, edema of the tegument, rupture of many tubercles with vacuolation and complete loss of spines. All infected treated mice groups, in comparison to positive control G2, showed a significant reduction in IL-4, IL-10, and TNF-α levels (*p*-value < 0.001), especially groups G6 and G7 (*p*-value < 0.05); both G6 and G7 values were nearer to the normal that indicated recovery of the liver tissue.

## 1. Introduction

Schistosomiasis is caused by worms of genus *Schistosoma* and is reported as one of the neglected parasitic tropical illnesses [1]. It has been reported in nearly seventy-eight countries, with about 236 million populations in 2019 needing treatment [2]. This disease predominantly affects rural communities as it is linked to poor sanitation, generally poor living conditions, and a lack of effective health policies [3]. Schistosomiasis control strategy mainly depends on treating infected individuals with PZQ [4]. The drawback of PZQ is that if a patient has a parasite at different life stages they will suffer from the disease symptoms regardless of the treatment [5].

Particularly in rural areas of Africa, over 80% of the population typically depends on herbal medicines. Recently, the potential design of new therapeutics has received marked attention because of their effectiveness against different parasitic infections [6]. 

Natural products and compounds derived from them exhibited urgency for developing new anti-schistosomiasis drugs over the past few years [7]. The anti-schistosomiasis efficiency of novel compounds is well elaborated using different levels like prophylactic strategies, killing the cercariae, schistosomula, and the adult parasite, and suppressive strategies such as the prohibition of worm egg-laying [8]. 

Ginger is one of the natural products that was used in traditional medicine as an anti-flatulent or carminative; the Greek physician Galen utilized ginger for the purification of the body [9]. Recent research showed that it has anticancer, antioxidant, anti-inflammatory, anti-hyperglycemic, antiapoptotic, anti-emetic, and antihyperlipidemic actions [10]. The few investigations that were made on the anti-helminthic activity of ginger and its phyto-constituents reported that both dried ginger aqueous extract and crude powder showed anti-helminthic activity in sheep [11]. Mostafa et al. investigated the effect of ginger ethyl acetate extract on the viability of *Schistosoma mansoni* adult pairs, cultured in vitro and in vivo in mice, and revealed that histopathological investigation of the intestine and liver of the ginger-treated mice showed smaller and fewer granulomas than in non-treated group [4].

Nanotechnology has promise for medication and nutrition because materials at the nanometres dimension gain novel properties different from those of both bulk material and isolated atoms [12]. Nano-materials can be used in nano-medicine for medical purposes in three different areas: nano-diagnosis, regenerative medicine, and controlled drug delivery [13]. Given the previous layout, the current study was designed to study the antischistosomal effect of ginger extract loaded on chitosan nanoparticles on *Schistosoma mansoni* experimentally infected mice, as a continued experiment from our previous work on this extract that gave promising results at the level of parasitological and biochemical parameters.

## 2. Materials and Methods

### 2.1. Preparation of Chitosan Nanoparticles (CsNPs)

**Figure 1:**
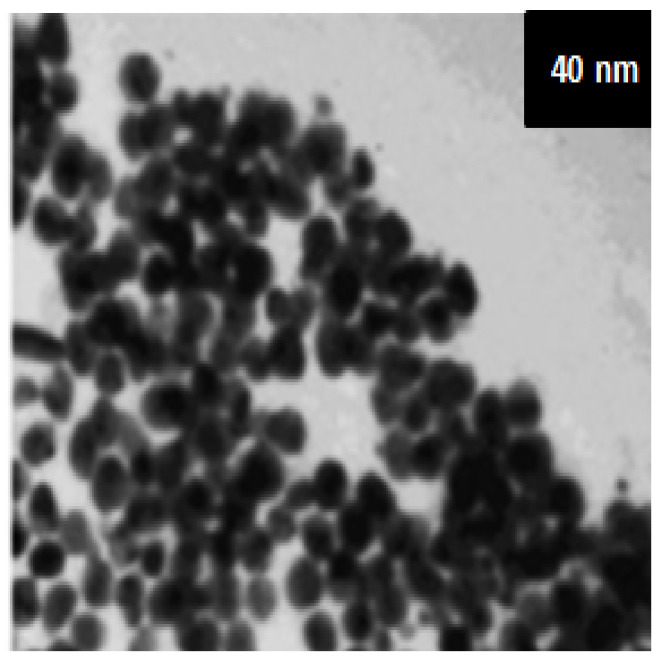
SEM micrograph of the synthesized CsNPs (Mean size = 60.08 nm).

Chitosan (85–93% deacetylation degree, Sigma Aldrich, St. Louis, MO, USA (CAS number: 9012-76-4)). Chitosan nanoparticles (CsNps) were synthesized via the ionotropic gelation of chitosan with sodium tripolyphosphate (TPP) anions as previously stated [14]. CsNps were prepared via the interaction of the opposite-charged macromolecules. Often, TPP is utilized for preparing CsNps because it is multivalent, non-toxic, and can produce gels by ionic interactions. The interaction is controlled by the charge density of chitosan and TPP that is relied on for the solution pH. CsNps were simultaneously fabricated with the drop-wise addition of chitosan solution (5 mL) to TPP (2 mL) solution at room temperature with magnetic stirring (1000× *g* for 1 h), to create an opalescent suspension. The NPs were segregated by centrifugation (20,000× *g* for 30 min). CsNps were freeze-dried and stored at 5 ± 3 °C. The freeze-dried NPs were weighed using viscometry (Figure 1).

### 2.2. Preparation of Ginger-Loaded Chitosan Nanoparticles (GCsNPs)

GCsNPs were prepared according to Dounighi et al. [15]. Two mL TPP solution containing either 250 mg or 500 mg of ginger, respectively, were added to 3 mg Cs NPs for each. Ginger-loaded chitosan NPs were segregated from aqueous suspension by centrifugation (20,000× *g* for 30 min). The free ginger in the collected supernatant was estimated spectrophotometrically using the Bradford protein assay at 595 nm. The ginger EE (encapsulation efficacy) and LC (loading capacity) of the NPs were assessed as follows:

%EE = [(A − B)/A] × 100

%LC = [(A − B)/C] × 100

where A is the total amount of ginger, B is the free amount of ginger and C is the weight of chitosan nanoparticles.

### 2.3. Experimental Design 

The present study was carried out on 68 female BALB/C mice (18–20 g, 6–8 weeks old), which were divided into 7 main groups (12 mice in each):

G1: uninfected, untreated.
G2: infected, untreated.
G3: infected/treated by praziquantel (PZQ, 500 mg/kg/day for 2 consecutive days).
G4: infected/treated by ginger extract (500 mg/kg).
G5: infected/treated by chitosan nanoparticles (3 mg/kg).
G6: infected/treated ginger (250 mg/kg) loaded chitosan nanoparticles.
G7: infected/treated ginger (500 mg/kg) loaded chitosan nanoparticles.

### 2.4. Infection of Mice 

Mice were infected with 80 ± 10 *S. mansoni* cercariae via the subcutaneous route (Peters and Warren, 1969). Mice and cercariae were purchased from the Schistosoma Biological Supply Program Unit, Theodor Bilharz Research Institute (TBRI), Imbaba, Giza, Egypt.

### 2.5. Preparation of Drugs and Mice Treatment

Aqueous extract of ginger was prepared by mixing ginger powder (30 g) in distilled water (60 mL), then it was squeezed through a cloth piece. The extract was stored at −20 °C till used, and freshly prepared every 3 days. Oral administration, ginger aqueous extract (500 mg/kg/day, 3 days/week for 5 weeks) with an esophageal tube starting from the 5th week post-infection [4]. Praziquantel tablets (Distocide 600 mg each, EIPICO/El-Asher Men Ramadan/Egypt) were crushed and administered orally as a suspension in cremophore-E1 (2%, Sigma-Aldrich Co., St. Louis, MO, USA) to mice (dose 500 mg/kg/b. wt.) for 2 consecutive days from the 5th week post-infection [16]. Chitosan (with a degree of deacetylation 85–93%) and TPP (Na tripolyphosphate) were procured from Sigma Aldrich (St. Louis, MO, USA). Chitosan nanoparticles were synthesized via the ionotropic gelation of chitosan with TPP anions. 

Mice were sacrificed by decapitation without anesthesia to avoid hepatic shift of the worms and separation of the copula due to the paralytic effect of anesthesia on worm musculature. Sacrification of all mice was done between 70 and 73 days post infection. Different parameters were used to determine the therapeutic effect of different doses of ginger extract loaded on chitosan nanoparticles (250 mg/kg and 500 mg/kg), in comparison to conventionally used Praziquantel (500 mg/kg/day for 2 consecutive days)for histopathological parameters: stained liver sections of experimental mice were used for studying the possible histopathological changes, Scanning Electron Microscopy; topographic visualization of isolated worms and Immunological parameters; determination of interleukins 4 and 10 (IL-4 and IL-10) and tumor necrosis factor-α (TNF-α) serum levels.

Blood samples were collected and allowed to sit for a minimum of 10 min (to clot). Then, they were centrifuged (3000× *g*, 10 min) and the serum was tipped off into a separate vial for biochemical and immunological parameters. Livers were collected and divided into pieces. A part of the left lobe of the liver was preserved in 10% buffered formalin and then processed into paraffin blocks. Some 4 μm-thick sections were serially cut, mounted on slides, and stained with Hematoxylin and Eosin (H&E) [17] and Masson trichrome (MT) stains. 

### 2.6. Microscopic Examination

It was done for all groups; sections stained with H&E were examined microscopically to show the histopathological changes before and after treatment. The number and diameter of granulomas were measured according to Abd El-Aal et al. [18].

#### 2.6.1. Number of Granulomas

The number of granulomas per low power field (10 × 10) was counted for each specimen, and for each specimen 5 fields were examined and the mean number was calculated. 

#### 2.6.2. Measurement of Granulomas 

Granulomas with single eggs in their centers and particularly in the lesions with great diameter were chosen. For each specimen, 5 fields were examined at lens power field (x) and the mean number was calculated in each group. The granuloma diameter of individual granulomas was measured using an ocular micrometer (Zeiss, Axioskop, Germany), which has graded lenses used to measure the diameter. The mean granulomas’ diameter was calculated and compared to the control and different groups.

#### 2.6.3. Types of Granulomas

Paraffin-embedded liver tissue sections were stained with Masson trichrome (MT) stain according to Kiernan [19], to show the density of fibrosis in granulomas that helps to determine the type of granulomas. Granulomas were classified as cellular, fibrocellular, and fibrous, according to the inflammatory cells (site and types) and the amount of collagen (stained bluish green by Masson trichrome) represented in the granulomas. For each specimen the percentage of each type of granuloma was determined.

### 2.7. Scanning Electron Microscopy (SEM) Examination

The SEM scan is a beam of electrons across the surface of a sample allowing visualization of the topography. Isolated worms were fixed in 2.5% glutaraldehyde for 2 h. The fixative was washed by keeping the worms at room temperature in 0.1% PBS (pH 7.4, for 24 h). They were placed into ascending grades of ethanol (30%, 50%, and 70%), each for 15 min using an automatic tissue processor (Leica EM TP). Worms were then dried in a CO_2_-critical point drier (Tousimis-Audosamdri-815). They were further coated with gold in a gold sputter coater (SPI-Module). The worms were examined by Scanning Electron Microscope (JEOL-JSM-5500 LV) using high-vacuum mode at the Regional Centre of Mycology and Biotechnology (Al-Azhar University, Cairo, Egypt).

### 2.8. Assessment of IL-4, IL-10, and TNF-α Levels

IL-4, IL-10, and TNF-α levels determination was done using sandwich ELISA [20]. The absorbance at 405 nm was estimated using ELISA micro-plate reader. The calculation of cytokine concentration from the standard curve utilizing Microplate Manager Software (Bio-Rad, Hercules, CA, USA) was carried out.

### 2.9. Statistics

Data are demonstrated as means ± SD (standard deviation). Variations among groups were assessed utilizing ANOVA, then the Student–Newman–Keuls *t*-test. The significance level was accepted with *p* ˂ 0.05.

## 3. Results

### 3.1. Histopathological Examination 

**Figure 2:**
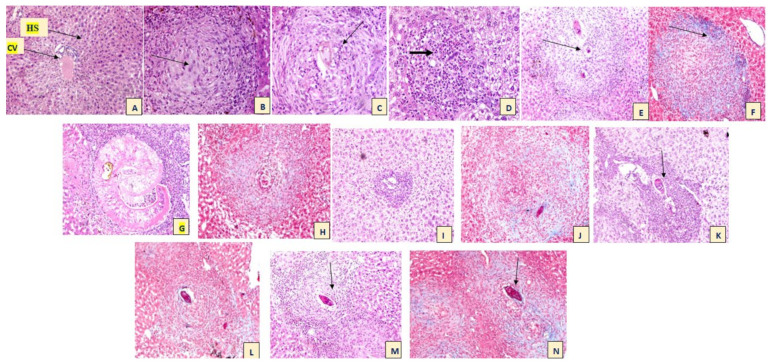
(**A**) Liver sections of a normal control mouse. CV: central vein—HS: hepatic sinusoid (H&E ×100); (**B**) Liver sections of positive control G2 showed fibrous granuloma with histiocytes infiltration (Arrow); (**C**) G2 showed fibrocellular granuloma surrounding intact ova (Arrow); (**D**) G2 showed cellular granuloma with eosinophils infiltration (Arrow) (H&E ×100); (**E**) Liver section of G3 granuloma showed a reaction around ova (Arrow) (H&E ×100); (**F**) G3 showed fibrocellular granuloma showing inflammatory cells (Arrowhead) collagen fibers (Arrow) (Masson trichrome stain ×400); (**G**) Liver sections of G4 showed bilharzial worm lodged in the portal tract surrounded by dense inflammatory cellular infiltrate rich in eosinophils (×200) (H&E); (**H**) G4 showed granulomatous reaction stained with Masson trichrome stain (×400); (**I**) Liver sections of G5 showed cellular granuloma formed of inflammatory cells mainly eosinophils centered in the portal area (H&E ×100); (**J**) G5 showed cellular granuloma formed of inflammatory cells (Masson Trichrome stain ×400); (**K**,**L**) Liver sections of G6 showed granuloma reaction around intact bilharzial ova in the portal tract (arrow) (H&E ×100 and Masson stain ×200, respectively); (**M**,**N**) Liver sections of G7 showed intact bilharzial ova (Arrow) surrounded by fibrocellular granulomatous reaction (H&E ×200 Masson trichrome stain ×400, respectively).

Liver sections of non-infected mice showed that hepatocytes were arranged in plates radially with respect to the central vein of each lobule. The place between the hepatocyte plates contained the hepatic sinusoids. The sinusoids were lined with endothelial cells which possess small lightly stained nuclei. All infected treated mice groups showed granulomatous reaction formation in their liver sections. Granuloma was made up of lymphocytes, eosinophils, histiocytes, macrophages, plasma cells, and fibroblasts surrounded by damaged bilharzial ova with disturbed liver architecture. Different types of granulomas are seen: cellular, fibrocellular, and fibrous. Fibrocellular granuloma was the most prominent type in all infected treated mice groups (Figure 2).

### 3.2. Hepatic Granuloma Types and Mean Count

**Figure 3:**
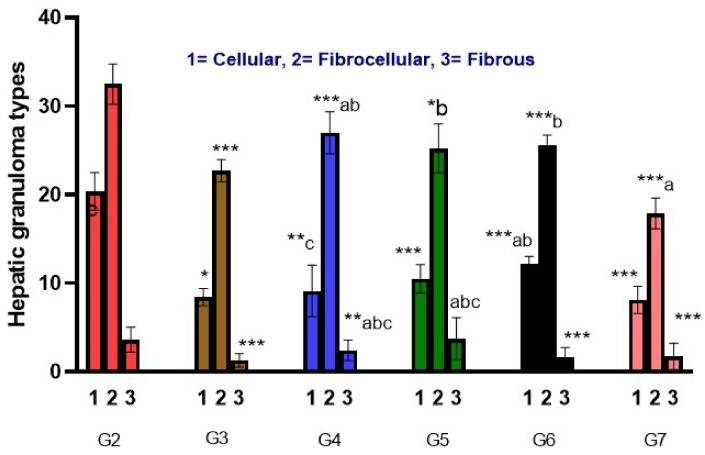
The effect of treatments on hepatic granuloma types and mean count. ANOVA (*p*-value ≤ 0.001), (*, **, ***) = statistically significant difference in comparison to G2 (*p*-value * < 0.05, ** < 0.01 and *** < 0.001), a = statistically significant difference in comparison to G3 (*p*-value <0.05), b = statistically significant difference in comparison to G7 (*p*-value < 0.05), c = statistically significant difference in comparison to G6 (*p*-value < 0.05).

**Figure 4:**
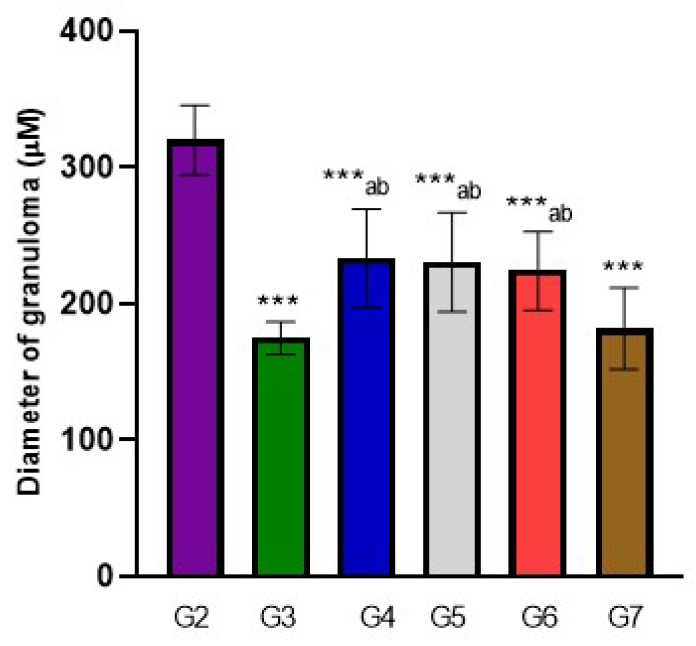
The effect of treatments on hepatic granuloma diameter. ANOVA (*p*-value ≤ 0.001), (***) = statistically significant difference in comparison to G2 (*p*-value, *** < 0.001), a = statistically significant difference in comparison to G3 (*p*-value < 0.05), b = statistically significant difference in comparison to G7 (*p*-value < 0.05).

There was a significant decrease in cellular granuloma count in all infected treated mice groups (*p*-value < 0.05), with the highest reduction in both G3 and G7 groups (8.43 ± 0.98 and 8.13 ± 1.55, respectively) followed by group G4 (9.14 ± 2.91) and G5 (10.50 ± 1.60) that showed a significant reduction in fibro-cellular granuloma in comparison to G7, and the least reduction in the count was noticed in group G6 (12.20 ± 0.84). On the other hand, Group G7 showed close results to PZQ in the mean count of cellular and fibrous granuloma followed by G5 (ginger alone 500 mg/kg); however, G6 showed close results to PZQ in the mean count of fibrocellular granuloma. Additionally, G7 versus G6 showed a remarkable decrease in the mean count of cellular and fibrocellular granuloma (*p*-value < 0.05), while an insignificant difference was noticed in the reduction in fibrous granuloma mean count between both groups. All infected treated mice groups showed a significant decrease in granuloma diameter in comparison to the infected non-treated group (*p*-value < 0.001). The greatest decrease in granuloma diameter was noticed in G3 (174.57 ± 12.1), followed by G7 (181.75 ± 29.9), and the least reduction was noticed in G4 (233.00 ± 36.24). Moreover, G7 showed similar results to G3 in granuloma diameter. Moreover, a noticeable reduction in the granuloma diameter of groups G7 and G3 versus G4, G5, and G6 (*p*-value < 0.05) was noticed (Tables S1 and S2 and Figure 3 and Figure 4).

### 3.3. Scanning Electron Microscopy (SEM) Results 

**Figure 5:**
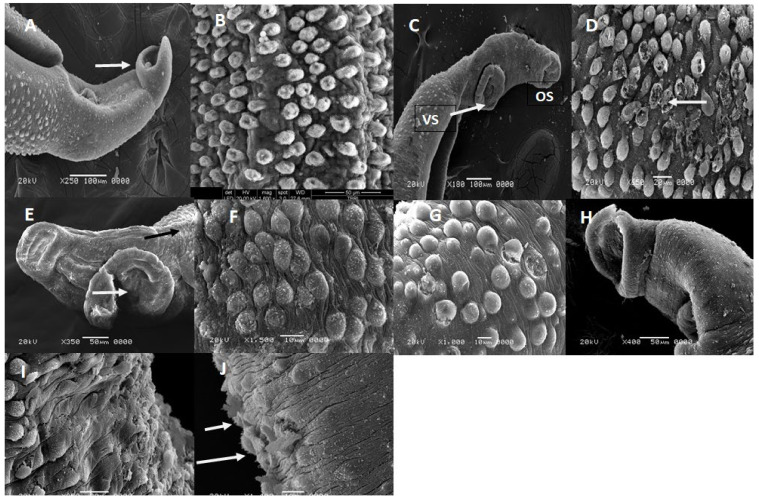
(**A**) SEM of *S. mansoni* recovered from positive control mouse showed the tegument with tubercles; (**B**) showed Edematous Oral sucker OS, retracted ventral suckers vs. (arrow) and the upper part of gynaecophoric canal (×1600 and ×250); (**C**) SEM of *S. mansoni* recovered from infected mouse treated by PZQ (500 mg/kg) (×180); (**D**) SEM of *S. mansoni* recovered from infected mouse treated by PZQ (500 mg/kg) showed ruptured vesicles (white arrow) (×650); (**E**) SEM of male *S. mansoni* recovered from ginger (500 mg/kg) treated mouse showed oedema, ulceration and cutting of ventral sucker (white arrow) with loss of spines (black arrow) (×350); (**F**) SEM of *S. mansoni* (dorso-lateral region) showed that wrinkles and ridges with invaginations and furrows and the coarse tubercles bear some spines (×1500); (**G**) SEM of *S. mansoni* adult recovered from chitosan nanoparticles 3 mg/kg treated mouse showed edematous tegument with formation of blebs and vesicles, rupture of some tubercles and loss of spines(×1000); (**H**) SEM of G6 showed abnormal edematous oral and retracted ventral sucker. Many sensory papillae are detected in the region above the ventral sucker (white arrow) (×400); (**I**,**J**) SEM of G7 showed oedema of the tegument, rupture of many tubercles (arrow) with vacuolation and complete loss of spines (×950 and ×1400).

An adult recovered from an infected mouse treated by PZQ (500 mg/kg) showed mild edema of oral and ventral suckers with some peeling and blebs around them and fissuring of the wall. Meanwhile, G6 and G7 showed rupture of numerous tubercles with a decrease in spines or complete loss of spines. Many holes are detected between the tubercles exposing the tegumental wrinkles (Figure 5).

### 3.4. Immunological Results

**Figure 6:**
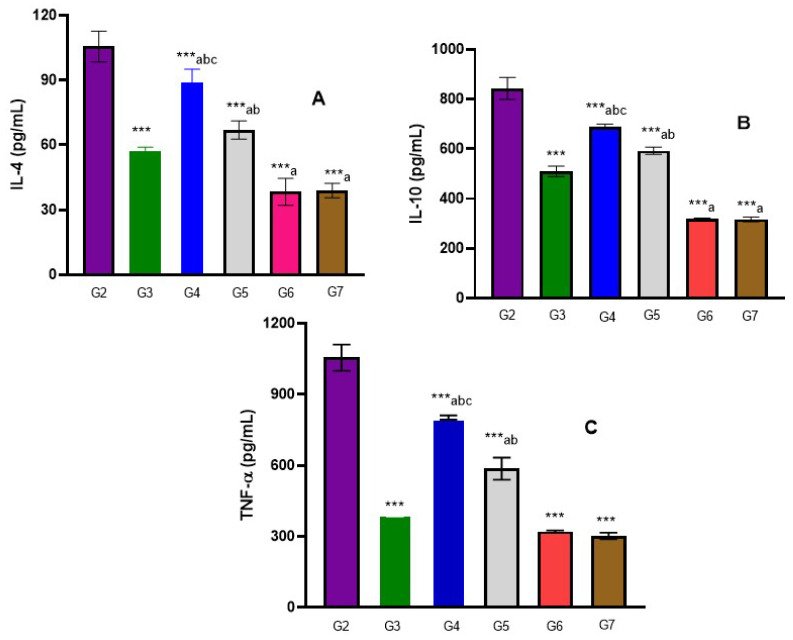
(**A**–**C**) Serum level of IL-4, IL_10 and TNF-α in different group. Mean ± SD (*p* value); ANOVA (*p*-value ≤ 0.001), (***) = statistically significant difference in comparison to G2 (*p*-value *** < 0.001), a = statistically significant difference in comparison to G3 (*p*-value < 0.05), b = statistically significant difference in comparison to G7 (*p*-value < 0.05), c = statistically significant difference in comparison to G6 (*p*-value < 0.05).

All infected treated mice groups showed a significant reduction in IL-4, IL-10 and TNF-α levels (*p*-value < 0.001) in comparison to positive control group G2, especially groups G6 and G7 (*p*-value < 0.05). Both G6 and G7 values were nearer to normal than other infected treated groups (Figure 6 and Table S3).

## 4. Discussion

Regarding morbidity and mortality rates, schistosomiasis is counted as the most serious helminthic disease of humanity. Vale et al. reported that >200 million people are infected worldwide, and 600 million are at risk of infection [21]. Estimates show that 220.8 million people (at least) need preventive treatment [2].

The most common species in Africa are *Schistosoma mansoni* and *S. haematobium* [22]. The medical sector’s reliance on a single drug for treating schistosomiasis and the failure of current measures to eradicate this disease led to great efforts to find a new anti-schistosomiasis drug [21,23]. Since schistosomiasis is one of the NTDs, the drug discovery channel is underfunded [24]. Natural products attracted great attention for therapeutic use for a long time, with 64% of all marketed drugs originating from natural products [25]. These natural products are a significant basis for discovering drugs because they have different phyto-constituents with various bioactivities [26].

Natural products are also odd as they are often rich in stereo-genic centers, and cover portions of chemical space that are usually not occupied by a greater part of synthetic drugs and medications [27]. One of the viable and intriguing research directions is the examination of medicinal plants as a novel technique for the attempted management of schistosomiasis. However, a lot of bioactive components from plants with schistosomicidal effects have been examined in various studies, notably those found in traditional herbal therapy [28].

Ginger is abundant in active constituents such as phenolic derivatives and terpenes [29]. These phenolic compounds include shogaols, gingerols, and paradols, as well as quercetin, zingerone, gingerenone-A, and 6-dehydrogingerdion [30]. In fresh ginger, gingerols are the major phenolics (e.g., 6-gingerol, 8-gingerol, and 10-gingerol) [31]. The anti-schistosome potential of ginger could be attributed to its phenolic constituents, particularly shogaols and gingerols that were reported to display remarkable antioxidant, antilarval, and anti-inflammation capacities [32,33,34].

Different investigators claimed that ginger had varying degrees of anti-schistosomal effects, either by speeding up the clearance of schistosome worms or by lowering the production of *S. mansoni* eggs and the size of liver granulomas [4]. Amaral et al. suggested that reduced synthesis of soluble egg antigens and/or inactivation of delayed hypersensitivity T cells, which control the inflammatory response and granulomatous development, may be the causes of ginger’s ability to reduce granulomatous formation [35].

Mostafa et al. assessed the anti-schistosomal potential of *Z. officinale* on *S. mansoni* and demonstrated the potency of this plant [4]. Although successful in vitro studies of the anti-schistosomal effectiveness of *Z. officinale* were done, these researchers reported no remarkable variation in treated and untreated mice for the in vivo experiments [36]. At its maximum nontoxic dose, the cytotoxic assay revealed that treated Vero cells did not possess any morphological differences comparing to the control, at the value of 250 μL/mL [37].

Drug release from natural polymers takes place relatively quickly from NPs because they decompose within a few hours. In contrast, synthetic polymers provide prolonged drug release because they can withstand degradation in the body for long periods of days, or even weeks [38]. The manufacture of NPs is providing stability or adequacy of functional groups and ease of functionalization [39,40]. NPs, due to their small size that ranges from 1 to 100 nm, can penetrate the very small capillaries throughout the body causing better solubility, absorption, and uptake and preventing the enzymatic degradation of labile drugs in the gastrointestinal tract. Moreover, nanoparticles highly aggregate more than normal drugs in the targeted tissues, typically contributing to decreased systemic toxicity [12,41]. CsNPs have a good affinity for negatively charged bioactive molecules such as antigens, antibodies, enzymes, cytokines, and polyanionic polymers, as they form ionic connections with endothelial cells that allow medicines to penetrate the biological barriers through adsorptive transcytosis [42,43]. CsNPs are reported to have better permeation and mucoadhesive-enhancing characters and promote absorption of the drug in the GI tract proximal part. Additionally, they inhibited some transporter proteins on the membrane of intestinal epithelial cells or enterocytes, which act as efflux pumps of drugs and contribute to drug resistance mechanisms [44].

Biodegradation has a major contribution in the metabolic fate of chitosan in the body. Enzymatically, Cs can be degraded by enzymes that can hydrolyze glucosamine-N-acetyl-glucosamine, glucosamine–glucosamine, and N-acetyl-glucosamine-N-acetyl-glucosamine linkages [45]. Cs is also known to be predominantly degraded in vertebrates by certain bacterial enzymes (chitinases) in the colon and by lysozyme [46]. Its extent and rate of biodegradability are dependent on DD (degree of deacetylation) [47]. Basically, given adequate time and appropriate conditions, chitosan, in most cases, would degrade sufficiently to be excreted [45]. Banerjee et al.’s investigation of CsNPs biodistribution revealed that these NPs are RES (reticuloendothelial system) evading and circulating in the blood for a remarkable period [48]. Many studies reported that the liver was the significant site of accumulation due to the take up by the Kupffer cells [49].

The present study showed a significant decrease in cellular granuloma count in all infected treated mice groups (*p*-value < 0.05), with the highest reduction in both G3 and G7 groups (8.43 ± 0.98 and 8.13 ± 1.55), respectively, followed by group G4 (9.14 ± 2.91); G5 exhibited notable reduction in fibrocellular granuloma in comparison to G7 and the least reduction in the count was noticed in group G6 (12.20 ± 0.84). However, G7 showed similar results to the group treated with PZQ (G3) in the mean count of cellular and fibrous granuloma, followed by G5 (ginger alone 500 mg/kg; G6 showed similar results to the PZQ group in the mean count of fibrocellular granuloma. G7 versus G6 showed a significant decrease in the mean count of cellular and fibrocellular granuloma (*p*-value < 0.05), while an insignificant difference was noticed in the reduction in fibrous granuloma mean count between both groups. All infected treated mice groups showed a significant decrease in granuloma diameter in comparison to the infected non-treated group (*p*-value < 0.001). The greatest decrease in granuloma diameter was noticed in G3 (174.57 ± 12.1) followed by G7 (181.75 ± 29.9), and the least reduction was noticed in G4 (233.00 ± 36.24). G7 showed similar results to G3 in granuloma diameter; also, a significant reduction in the granuloma diameter of G7 and G3 versus G4, G5, and G6 (*p*-value < 0.05) was noticed.

There was a restoration of normal hepatocyte appearance and normal hepatic strand organization by GCs Nps 500 mg/kg treatment, which may be due to amelioration and restoration of the level of acidic, basic, and neutral proteins as well as carbohydrate contents, and by scavenging free radicals and the potent antioxidant effect. These results were similar to results recorded by previous researchers [50].

Our results showed a reduction in the mean granuloma size and count by ginger 500 mg/kg CsNps treatment similar to that of PZQ 500 mg/kg with insignificant difference, which may be due to suppression of Th1 and Th2 lymphocytes and their cytokines that mediated granuloma formation. Contrary to our results, Elbaz and Esmat reported that liver lesions appeared to regress more rapidly after Praziquantel than after treatment with other schistosomicidal drugs [51].

Mahmoud et al. revealed anti-schistosomal effects of *Z. officinale* with regard to the histopathological changes observed in the small intestines of infected mice [52]. Aly and Mantawy observed, as a result of a sharp decline in granulomas and retraction granuloma formation, that most eggs were retained inside intestinal compartments without triggering an inflammatory response [53]. By scavenging free radicals with its potent antioxidant activity, Z. officinale also helped to restore the appearance of normal hepatocytes and normal hepatic strand organization [54]. El-derbawy et al. demonstrated that *S. mansoni*-infected mice treated with aqueous ginger extract loaded on chitosan nanoparticles showed an apparent decrease in elevated liver peroxidation and improved liver function that reflects the antioxidant defense system [55]. Another study reported the significant synergistic effect of ginger-derived nanoparticles when used either alone or combined with PZQ or mefloquine, as liver granuloma diameter and number significantly decreased and the type of granuloma became more fibro-cellular [54].

Since the tegument of *Schistosoma* is an essential target for different medications, various researchers exploited changes in the surface ultrastructure (scanning electron microscopy, SEM) of *Schistosoma worms* for this purpose [56,57]. It was demonstrated that the worms’ tegumental modification was a substantial aspect of drug capacity that caused death and worms’ elimination with a stop of egg production [58]. Our results showed that adult worms recovered from an infected mouse treated by PZQ (500 mg/kg) showed mild edema of oral and ventral suckers with some peeling and blebs around them and fissuring of the wall. Meanwhile, G6 and G7 showed rupture of numerous tubercles with a decrease in spines or complete loss of spines. Many holes are detected between the tubercles with exposing the tegumental wrinkles. The current results harmonized with those noted by Shereen et al., who proved that chitosan improved *Nigella sativa* effectiveness on *S. mansoni* adult and the morphological variations on male worm surfaces were swollen suckers and worm deformity [59]. The teguments were inflated in some areas and flattened in other portions with shrinkage and wrinkling and oedematous inter-papillary ridges, whereas female teguments revealed remarkable deformation that appeared as shrinking and furrowing. Additionally, the surface topography displayed that the male worms obtained from Sidr honey-treated mice possessed comprehensive loss of spines [57]. Supporting our results, Sadek et al. reported that Cs Nps treatment either alone or loaded with PZQ led to adult changes in the form of edema and whole tegument swelling [60].

The results are also in line with former studies that reported the appearance of tubercles edema, swelling, and dilatation of the adult male *Schistosoma* suckers treated with *N. sativa*-loaded Cs Nps [61].

It is noteworthy that the ginger-killing potential to worms could be due to its produced morphological variations. Its induced harm to suckers resulted in a loss of worm adherence capability to blood vessels, leading to more difficulty in the nutrients ingestion from the blood. Besides, the ginger-caused deleterious effect on the teguments along with the worm’s body would impair the tegument functioning and devastate the worm defense system, thus it could readily be attacked by the host immune system [62].

The current study also showed that all infected-treated mice groups revealed a significant reduction in IL-4, IL-10 and TNF-α levels (*p*-value < 0.001) in comparison to positive control group G2, and especially groups G6 and G7 (*p*-value < 0.05). Both G6 and G7 values were nearer to normal than other infected treated groups, and there was an insignificant difference between PZQ 500 mg/kg (Group 3), ginger 250 mg/kg and ginger Cs Nps 500 (Groups 6 and 7) in decreasing the production of inflammatory mediator TNF-α.

IL-10 is substantial for keeping a non-lethal chronic infection and reducing the parasite’s egg-induced hepatocyte injury. Skin resident tissue macrophages, which face *S. mansoni* secretory/excretory products through infection, produce IL-10 in vivo early post-infection with *S. mansoni* cercariae [53].

Infected mice treated with ginger Cs Nps 500 mg/kg possessed a lessening in the IL-4 and IL-10 levels, which agreed with the report stating that ginger extract declined the inflammation mediators that have a marked contribution in schistosomal-liver fibrosis and its consequences [53]. Moreover, a lessening in the IL-10 level in PZQ-treated infected mice, comparing to the infected group, was in alignment with the study of Brown et al. (2005); however, they are contrary to Wilson et al. (2011) who reported an elevation in IL-4 and IL-10 after PZQ usage, and Aly et al. declared that the IL-10 elevation with PZQ treating may diminish the granuloma size [63,64,65].

Besides, Hassan et al. demonstrated that IL-4 and IL-10 were raised subsequently to *S. mansoni* infection, which was in accordance with the current results [66]. Large amounts of IL-4 are produced in *S. mansoni*-infected mice and are proposed to be responsible for granuloma formation, and cytokine IL-10 is fundamental for generating host-protective homeostatic circumstances in schistosomiasis. TNF-α, a pro-inflammation cytokine, is considered a potential mediator of macrophage recruitment and is implicated in inflammatory reactions up-regulation, and positively affects granuloma formation around Schistosome eggs [67]. The oxidative stress reduction and inflammation risk factors modulation may be considered targets for molecular interventions for treating liver fibrosis and its complications in murine schistosomiasis [53].

## 5. Conclusions

The improvement in the immune response could be assigned to the immuno-stimulant potential of chitosan or be due to its antimicrobial and anti-inflammatory capacity. Accordingly, combination therapy of ginger and chitosan nanoparticles could lead to a quick recovery, lesser side effects, and lower drug doses; in addition, it resulted in a reduction in the number and size of granulomas and restoration of liver function to normal [67]. Based on our findings, Ginger loaded on chitosan NPs have a promising antischistosomal effect, ameliorating the host inflammatory response. In addition to its influence on *S. mansoni* worms, it retained normal hepatic organization and had a hepatoprotective effect that may contribute to reducing chronic schistosomiasis complications. Further, the expansion of this investigation to human trials for assessing the therapeutic potential of Ginger loaded on chitosan NPs for schistosomiasis is recommended.

## Data Availability

Not applicable.

## Supplementary Materials

The following supporting information can be downloaded at: https://www.mdpi.com/article/10.3390/life12111834/s1, Table S1. The effect of different used treatments on hepatic granuloma types and mean count in *S. mansoni* infected group; Table S2. The effect of different treatments on hepatic granuloma diameter (um); Table S3. Serum level of IL-4, IL-10, and TNF-α in different group.
