# Supplementary material for: In Vivo Evaluation of the Anti-Schistosomal Potential of Ginger-Loaded Chitosan Nanoparticles on Schistosoma mansoni: Histopathological, Ultrastructural, and Immunological Changes
Source: Life (Basel). 2022 Nov 9;12(11):1834. doi: 10.3390/life12111834 (PMC9696985; doi:10.3390/life12111834)
Supplement: Supplementary file 1 [file life-12-01834-s001.zip › life-1970062-supplementary.pdf]

Table S1. The **effect** of different used treatments on hepatic granuloma types and mean count in *S. mansoni* infected group.

Group	Cellular	Fibrocellular	fibrous
**2**	20.38 ± 2.13	32.50 ± 2.27	3.63 ± 1.41
**3**	8.43 ± 0.98<br>(0.004*)	22.71 ± 1.25<br>(0.001***)	1.29 ± 0.76<br>(0.001***)
**4**	9.14 ± 2.91^{c}<br>(0.009**)	27.00 ± 2.38^{ab}<br>(0.001***)	2.43 ± 1.13^{abc}<br>(0.01**)
**5**	10.50 ± 1.60<br>(0.001***)	25.25 ± 2.76^{b}<br>(0.001*)	3.75 ± 2.38^{abc}<br>(0.063)
**6**	12.20 ± 0.84^{ab}<br>(0.001***)	25.60 ± 1.14^{b}<br>(0.001***)	1.60 ± 1.14<br>(0.001***)
**7**	8.13 ± 1.55<br>(0.001***)	17.88 ± 1.73^{a}<br>(0.001***)	1.75 ± 1.49<br>(0.001***)

ANOVA (p-value≤0.001), (*, **, ***) =statistically significant difference in comparison to G2 (P-value *<0.05, **<0.01 and ***<0.001), a =statistically significant difference in comparison to G3 (P-value <0.05), b =statistically significant difference in comparison to G7 (P-value <0.05), c= statistically significant difference in comparison to G6 (P-value <0.05).

Table S2. The effect of different treatments on hepatic granuloma diameter (um).

Group	Diameter of granuloma(μm)
**2**	320.00 ± 25.6
**3**	174.57 ± 12.1<br>(0.001***)
**4**	233.00 ±36.24^{ab}<br>(0.001***)
**5**	230.25 ± 36.3^{ab}<br>(0.001***)
**6**	224.00 ± 28.8^{ab}<br>(0.001***)
**7**	181.75 ± 29.9<br>(0.001***)

ANOVA (p-value≤0.001), (*, **, ***) =statistically significant difference in comparison to G2 (P-value *<0.05, **<0.01 and ***<0.001), a =statistically significant difference in comparison to G3 (P-value <0.05), b =statistically significant difference in comparison to G7 (P-value <0.05), c= statistically significant difference in comparison to G6 (P-value <0.05).

Table S3. Serum level of IL-4, IL-10, and TNF-α in different group.

Group	IL- 4 pg/mL	IL- 10 pg/mL	TNF-α pg/mL
**2**	105.50 ± 7.09	843.50 ± 44.23	1054.88 ± 55.62
**3**	57.00 ± 2.00^{d}<br>(0.001***)	510.43 ± 20.69^{d}<br>(0.001***)	396.00 ± 11.43^{d}<br>(0.001***)
**4**	91.00 ± 4.04^{abcd}	689.86 ± 10.22^{abcd}	802.29 ± 8.75^{abcd}

	(0.001***)	(0.001***)	(0.001***)
**5**	66.88 ± 4.16^{abcd}<br>(0.001***)	593.63 ± 14.21^{abcd}<br>(0.001***)	586.50 ± 46.54^{abcd}<br>(0.001***)
**6**	38.40 ± 6.23^{ad}<br>(0.001***)	317.60 ± 4.93^{ad}<br>(0.001***)	320.80 ± 4.49^{d}<br>(0.001***)
**7**	39.00 ± 3.30^{ad}<br>(0.001***)	316.88 ± 9.14^{ad}<br>(0.001***)	302.13 ± 14.08^{d}<br>(0.001***)

Mean ± SD (P value); ANOVA (p-value≤0.001), (*, **, ***) =statistically significant difference in comparison to G2 (P-value *<0.05, **<0.01 and ***<0.001), a =statistically significant difference in comparison to G3 (P-value <0.05),b =statistically significant difference in comparison to G7 (P-value <0.05),c= statistically significant difference in comparison to G6 (P-value <0.05).
